# Supplementary material for: Characterising the hippocampal response to perception, construction and complexity
Source: Cortex. 2021 Apr;137:1–17. doi: 10.1016/j.cortex.2020.12.018 (PMC8048772; doi:10.1016/j.cortex.2020.12.018)
Supplement: Multimedia component 1 [file mmc1.docx]

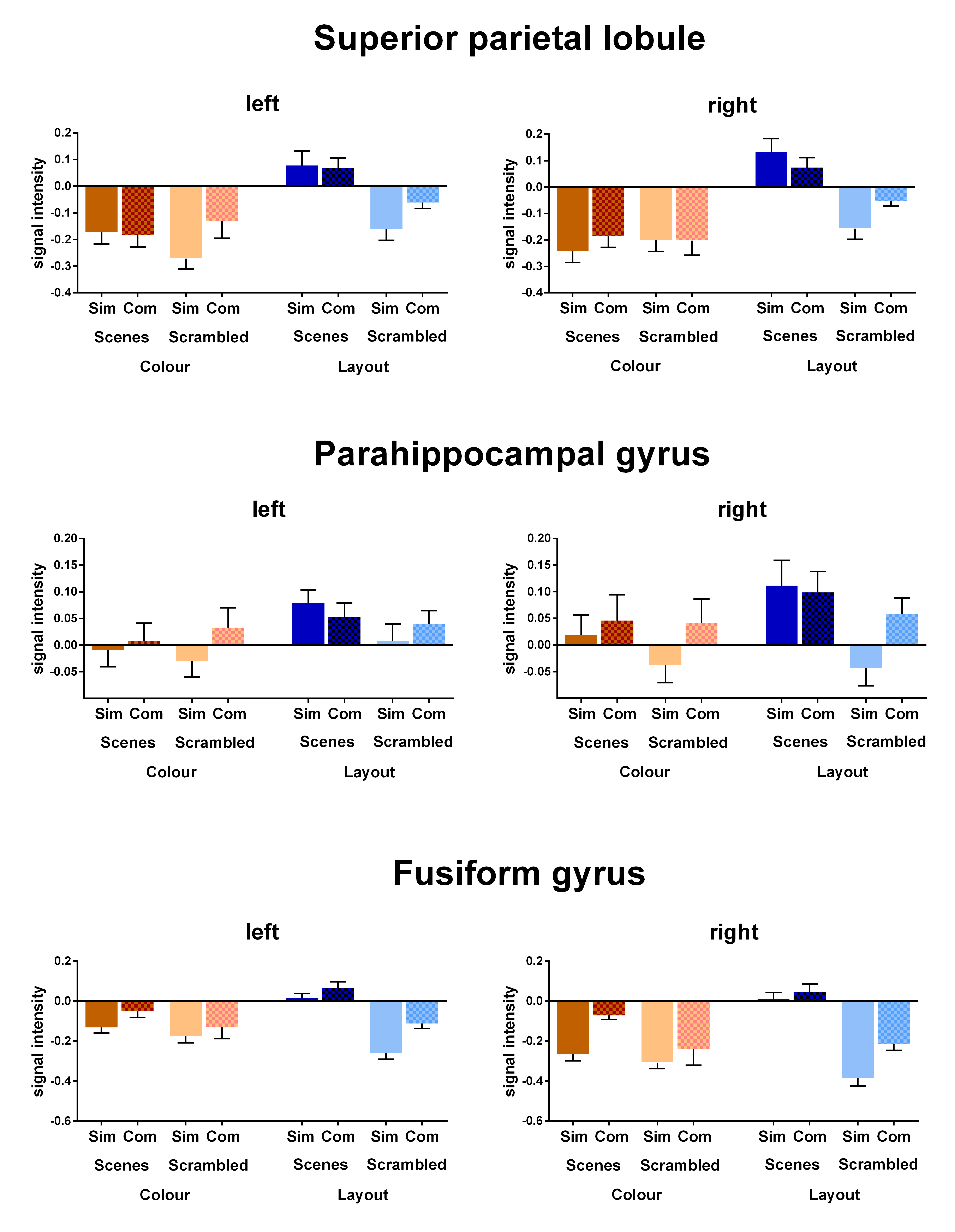
**Supplementary Material**

**Figure S1. Additional signal intensities**

Additional signal intensities extracted from brain regions associated with scene processing in the contrast driven PLS #1 analysis, namely the superior parietal lobule (MNI: left -20 -60 56; right 20 -60 56), parahippocampal gyrus (left -20 -15 -25; right 28 -8 -30), and fusiform gyrus (left -28 -42 -10; right 28 -42 -10). Bar graphs depict means and standard errors of the eight conditions. Sim=simple, com=complex. Of note, signal intensities are compared to an arbitrary fMRI baseline, hence negative values do not necessarily represent deactivations.

**Table S1: Overview of the strategies used*.**

| **Participant #** | **Colour conditions - simple and complex, scenes and scrambled** |
| --- | --- |
| S1 | looked at the centre of the image, compared bright areas between images |
| S2 | looked at blobs of colour, compared parts of the image but not related to the scene content |
| S3 | compared bright parts of the images |
| S4 | compared bright parts of the images |
| S5 | looked at blobs of colour, compared parts of the image but not related to the scene content |
| S6 | examined the colour along the borders of the images |
| S7 | tried to see both images at the same time |
| S8 | looked at blobs of colour, compared parts of the image but not related to the scene content |
| S9 | looked at blobs of colour, compared parts of the image but not related to the scene content |
| S10 | looked at blobs of colour, compared parts of the image but not related to the scene content |
| S11 | focused on the big picture |
| S12 | looked at blobs of colour, compared parts of the image but not related to the scene content |
| S13 | looked at blobs of colour, compared parts of the image but not related to the scene content |
| S14 | looked at blobs of colour, compared parts of the image but not related to the scene content |
| S15 | looked at blobs of colour, compared parts of the image but not related to the scene content |
| S16 | kept comparing global differences in the images |
| S17 | looked at blobs of colour, compared parts of the image but not related to the scene content |
| S18 | looked at the whole image |
| S19 | stared in the middle and so could see both images |
| S20 | looked from a distance at the whole image |
|  |  |
| **Participant #** | **Layout conditions - simple and complex, scenes and scrambled** |
| S1 | examined relationships between image features |
| S2 | examined relationships, focus on scenes |
| S3 | examined distance of objects from the edges |
| S4 | examined relationships between objects |
| S5 | looked at relationships, tried to see the layout of the whole picture |
| S6 | Focused on relationships between objects in scenes |
| S7 | focused on relationships between features within and between images |
| S8 | focused on relationships between features within and between images |
| S9 | focused on relationships between features within and between images |
| S10 | focused on relationships between features within and between images |
| S11 | focused on the spatial details |
| S12 | focused on relationships between features within and between images |
| S13 | focused on relationships between features within and between images |
| S14 | focused on relationships between features within and between images |
| S15 | focused on relationships between features within and between images |
| S16 | scanned around the image to see the layout |
| S17 | focused on relationships between features within and between images |
| S18 | identified most significant object, then examined their spatial relationships |
| S19 | looked horizontally and vertically, back and forth between images |
| S20 | measured and compared distance between objects |

*Note that participants did not describe different strategies for scenes or scrambled, simple or complex images

**Table S2: Peak coordinates of the data driven PLS LV1**

| **Region** | **side** | **MNI coordinates** | | | **BSR** |
| --- | --- | --- | --- | --- | --- |
|  |  | **X** | **Y** | **Z** |  |
| **Scene construction** | |  |  |  |  |
| Middle occipital gyrus | left | -40 | -84 | 0 | 14.60 |
| Parahippocampal gyrus* | left | -30 | -30 | -20 | 9.96 |
| Precuneus* | left | -8 | -64 | 58 | 5.14 |
| Inferior partietal lobule* | left | -52 | -38 | 48 | 4.23 |
| Middle occipital gyrus | right | 36 | -82 | -6 | 14.3 |
| Fusiform gyrus* | right | 30 | -44 | -16 | 12.5 |
| Precuneus* | right | 14 | -44 | 42 | 2.92 |
| Inferior parietal lobule* | right | 50 | -44 | 50 | 2.67 |
| Inferior frontal gyrus | right | 46 | 10 | 28 | 7.84 |
| Superior parietal lobule* | right | 20 | -60 | -56 | 5.53 |
| Hippocampus (anterior)* | right | 28 | -2 | -28 | 5.23 |
| Fusiform gyrus* | right | 28 | -42 | -10 | 5.10 |
| Hippocampus (anterior)* | left | -28 | -4 | -18 | 5.02 |
| Cerebellum | left | -14 | -46 | -48 | 4.87 |
| Cerebellum | right | 20 | -42 | -46 | 4.60 |
| Insula | right | 30 | 22 | 6 | 4.06 |
| Middle cingulate gyrus | right | 8 | -2 | 32 | 4.00 |
| Middle frontal gyrus | right | 38 | 62 | 10 | 3.98 |
| Superior parietal lobule* | left | -20 | -60 | 56 | 3.67 |
| Inferior frontal gyrus | left | -40 | 36 | -18 | 3.42 |
| Ventromedial prefrontal cortex | left | -4 | 36 | -18 | 3.33 |
| Parahippocampal gyrus* | right | 28 | -8 | -30 | 3.25 |
| Fusiform gyrus* | left | -28 | -42 | -10 | 3.05 |
|  |  |  |  |  |  |
| **All other conditions** | |  |  |  |  |
| Angular gyrus | left | -48 | -56 | 34 | 5.06 |
| Anterior cingulate gyrus | right | 4 | 32 | 0 | 4.64 |
| Angular gyrus | right | 54 | -60 | 36 | 4.55 |
| Brainstem | left | -8 | -38 | -48 | 4.27 |
| Cerebellum | right | 18 | -80 | -30 | 4.21 |
| Precuneus | left | -10 | -54 | 28 | 3.97 |
| Superior frontal gyrus | left | -12 | 36 | 48 | 3.26 |
| Middle temporal gyrus | left | -64 | -24 | -10 | 3.11 |
| Middle temporal gyrus | right | 60 | -20 | -18 | 2.88 |
| Middle cingulate gyrus |  | 0 | -22 | 40 | 2.58 |

X,Y, and Z coordinates in MNI space, BSR=Bootstrap ratio, *=regions included in larger clusters

**Table S3: Peak coordinates of the data driven PLS LV2**

| **Region** | **side** | **MNI coordinates** | | | **BSR** |
| --- | --- | --- | --- | --- | --- |
|  |  | **X** | **Y** | **Z** |  |
| **Visual simplicity** | |  |  |  |  |
| Calcarine gyrus | left | -6 | -92 | -4 | 12.42 |
| Postcentral gyrus | left | -54 | -6 | 48 | 4.63 |
| Middle cingulate gyrus | left | -6 | 14 | 40 | 4.21 |
| Precuneus | left | -10 | -56 | 20 | 3.95 |
| Middle frontal gyrus | left | -4 | -22 | 54 | 3.90 |
| Caudate | right | 10 | 2 | 6 | 3.81 |
| Thalamus | left | -16 | -16 | -2 | 3.65 |
| Cuneus | left | -16 | -70 | 26 | 3.30 |
| Middle cingulate gyrus | left | -12 | -28 | 38 | 3.20 |
| Precuneus | left | -10 | -38 | 54 | 3.11 |
|  |  |  |  |  |  |
| **Visual complexity** | |  |  |  |  |
| Inferior temporal gyrus | right | 52 | -54 | -18 | 6.20 |
| Inferior occipital gyrus* | right | 36 | -82 | -6 | 5.41 |
| Inferior frontal gyrus | right | 32 | 32 | 10 | 5.64 |
| Cerebellum | left | -20 | -64 | -34 | 5.56 |
| Middle temporal gyrus | left | -60 | -56 | -2 | 5.06 |
| Inferior temporal gyrus | left | -50 | -72 | -10 | 4.86 |
| Inferior occipital gyrus* | left | -38 | -82 | -4 | 3.21 |
| Inferior parietal lobule | right | 54 | -30 | 48 | 4.62 |
| Ventromedial prefrontal cortex | right | 16 | 42 | -18 | 4.21 |
| Pallidum | left | -26 | -12 | -4 | 3.79 |
| Fusiform gyrus | right | 28 | -4 | -36 | 3.66 |
| Middle frontal gyrus | right | 32 | 6 | 54 | 3.49 |
| Cerebellum | left | -4 | -56 | -58 | 3.46 |
| Superior frontal gyrus | right | 8 | 28 | 38 | 3.36 |
| Fusiform gyrus* | left | -36 | -45 | -8 | 3.25 |
| Middle frontal gyrus | right | 40 | 60 | 0 | 3.20 |
| Middle frontal gyrus | left | -36 | 56 | -4 | 3.08 |
| Cerebellum | left | -8 | -58 | -8 | 2.84 |
| Supramarginal gyrus | left | -62 | -20 | 24 | 2.71 |
| Inferior parietal lobule | left | -38 | -46 | 52 | 2.59 |

X,Y, and Z coordinates in MNI space, BSR=Bootstrap ratio, *=regions included in larger clusters

**Table S4: Peak coordinates of the data driven PLS LV3**

| **Region** | **side** | **MNI coordinates** | | | **BSR** |
| --- | --- | --- | --- | --- | --- |
|  |  | **X** | **Y** | **Z** |  |
| **Scene perception** | |  |  |  |  |
| Fusiform gyrus | right | 24 | -36 | -16 | 8.44 |
| Parahippocampal gyrus | left | -22 | -42 | -10 | 7.58 |
| Angular gyrus | left | -38 | -60 | 38 | 6.91 |
| Inferior frontal gyrus | right | 46 | 36 | 4 | 4.82 |
| Posterior cingulate gyrus | left | -2 | -36 | 40 | 4.65 |
| Hippocampus (posterior)* | left | -36 | -28 | -14 | 4.55 |
| Fusiform gyrus* | left | -36 | 54 | -10 | 4.27 |
| Supramarginal gyrus | left | -58 | -20 | 20 | 4.06 |
| Postcentral gyrus | left | -52 | -18 | 54 | 3.65 |
| Inferior occipital gyrus* | right | 38 | -76 | -8 | 3.52 |
| Inferior temporal gyrus | right | 34 | 0 | -38 | 3.42 |
| Inferior frontal gyrus | right | 58 | 12 | 10 | 3.20 |
| Inferior occipital gyrus* | left | -34 | -76 | -10 | 2.83 |
| Parahippocampal gyrus* | right | 28 | -26 | -20 | 2.57 |
| Hippocampus (posterior)* | right | 32 | -32 | -4 | 2.47 |
|  |  |  |  |  |  |
| **Scrambled construction** | |  |  |  |  |
| Dorsomedial prefrontal cortex | right | 12 | 38 | 22 | 6.52 |
| Superior parietal lobule | right | 16 | -70 | 58 | 5.30 |
| Cerebellum | right | 36 | -68 | -46 | 5.06 |
| Middle cingulate cortex | left | -8 | 14 | 44 | 4.80 |
| Inferior frontal gyrus | right | 54 | 10 | 24 | 4.79 |
| Brainstem | left | -4 | -18 | -12 | 4.46 |
| Middle temporal gyrus | right | 50 | -24 | -16 | 4.11 |
| Middle frontal gyrus | left | -36 | -2 | 44 | 4.11 |
| Superior parietal lobule | left | -18 | -56 | 60 | 3.96 |
| Cerebellum | left | -14 | -46 | -58 | 3.73 |
| Cerebellum | right | 6 | -58 | -52 | 3.72 |
| Inferior parietal lobule | right | 40 | -38 | 52 | 3.35 |
| Caudate | right | 10 | 6 | 8 | 3.12 |
| Postcentral gyrus | right | 66 | 0 | 30 | 2.60 |

X,Y, and Z coordinates in MNI space, BSR=Bootstrap ratio, *=regions included in larger clusters

**Table S5: Peak coordinates of the contrast driven PLS - scenes versus scrambled images**

| **Region** | **side** | **MNI coordinates** | | | **BSR** |
| --- | --- | --- | --- | --- | --- |
|  |  | **X** | **Y** | **Z** |  |
| **Naturalistic scenes** | |  |  |  |  |
| Middle occipital gyrus | left | -38 | -78 | 16 | 19.02 |
| Fusiform gyrus* | left | -30 | -44 | -16 | 15.13 |
| Lingual gyrus* | right | 26 | -64 | -12 | 12.29 |
| Lingual gyrus* | left | -24 | -62 | -10 | 11.31 |
| Parahippocampal gyrus* | right | 24 | -30 | -18 | 9.69 |
| Superior frontal gyrus | left | -24 | 12 | 58 | 6.80 |
| Fusiform gyrus | right | 32 | -2 | -36 | 6.03 |
| Precentral gyrus | right | 40 | 2 | 56 | 5.44 |
| Parahippocampal gyrus* | left | -36 | -18 | -22 | 4.84 |
| Middle frontal gyrus | right | 52 | 48 | -6 | 4.57 |
| Inferior parietal lobule* | right | 28 | -62 | 42 | 4.27 |
| Hippocampus (anterior)* | right | 18 | -8 | -22 | 4.27 |
| Hippocampus (posterior)* | right | 23 | -36 | 2 | 3.28 |
| Brainstem | left | -8 | -34 | -36 | 3.64 |
| Ventromedial prefrontal cortex | right | 2 | 50 | -22 | 3.63 |
| Middle cingulate gyrus | right | 8 | -2 | 32 | 3.58 |
| Middle frontal gyrus | left | -46 | 50 | -4 | 3.46 |
| Globus Pallidus | left | -14 | -2 | 4 | 3.42 |
| Cerebellum | left | -30 | -68 | -50 | 3.19 |
| Rolandic operculum | left | -50 | -22 | 18 | 3.01 |
| Superior temporal pole | right | 50 | 20 | -16 | 2.89 |
| Hippocampus (anterior)* | left | -24 | -10 | -20 | 2.71 |
| Hippocampus (posterior)* | left | -18 | -31 | -2 | 2.67 |
| Inferior parietal lobule* | left | -50 | -44 | 50 | 2.57 |
| Middle frontal gyrus | right | 26 | 36 | -14 | 2.52 |
|  |  |  |  |  |  |
| **Scrambled images** | |  |  |  |  |
| Precuneus | left | -14 | -54 | 30 | 4.31 |
| Anterior cingulate gyrus | right | 2 | 30 | 0 | 3.82 |
| Cerebellum | right | 2 | -60 | -48 | 3.71 |
| Calcarine gyrus | left | -12 | -92 | 4 | 3.49 |
| Middle frontal gyrus | right | 26 | 48 | 36 | 3.36 |
| Middle temporal gyrus | right | 52 | -28 | -10 | 3.34 |
| Middle cingulate gyrus | left | -2 | -24 | 30 | 2.99 |
| Superior frontal gyrus | right | 12 | 42 | 22 | 2.91 |
| Superior frontal gyrus | left | -6 | 60 | 8 | 2.32 |

X,Y, and Z coordinates in MNI space, BSR=Bootstrap ratio, *=regions included in larger clusters

**Table S6: Peak coordinates of the contrast driven PLS - scene construction versus scene perception**

| **Region** | **side** | **MNI coordinates** | | | **BSR** |
| --- | --- | --- | --- | --- | --- |
|  |  | **X** | **Y** | **Z** |  |
| **Scene construction** | |  |  |  |  |
| Superior parietal lobule | right | 16 | -74 | 58 | 8.16 |
| Precentral gyrus | right | 32 | 0 | 54 | 7.97 |
| Cerebellum | right | 36 | -70 | -46 | 6.33 |
| Fusiform gyrus* | right | 34 | -38 | -16 | 5.86 |
| Fusiform gyrus | left | -42 | -60 | -14 | 5.73 |
| Hippocampus (anterior) | left | -32 | -2 | -22 | 5.08 |
| Middle occipital gyrus* | right | 34 | -80 | 26 | 4.97 |
| Superior frontal gyrus | right | 6 | 62 | 36 | 4.89 |
| Lingual gyrus* | left | -24 | -48 | -10 | 4.61 |
| Superior occipital gyrus* | left | -28 | -82 | 34 | 4.34 |
| Cerebellum | left | -30 | -46 | -38 | 4.16 |
| Ventromedial prefrontal cortex | left | -8 | 44 | -22 | 4.08 |
| Hippocampus (anterior) | right | 24 | -8 | -24 | 3.81 |
| Parahippocampal gyrus* | right | 28 | -22 | -24 | 3.75 |
| Middle frontal gyrus | left | -38 | 36 | -16 | 3.54 |
| Parahippocampal gyrus* | left | -22 | -16 | -22 | 3.21 |
| Inferior frontal gyrus | right | 30 | 46 | -18 | 3.04 |
|  |  |  |  |  |  |
| **Scene perception** | |  |  |  |  |
| Angular gyrus | left | -48 | -60 | 42 | 6.60 |
| Anterior cingulate gyrus | right | 2 | 32 | 2 | 4.45 |
| Superior frontal gyrus | right | 18 | 30 | 38 | 3.91 |
| Angular gyrus | right | 54 | -54 | 50 | 3.89 |
| Precuneus | right | 14 | -50 | 34 | 3.72 |
| Hippocampus (posterior) | left | -28 | -36 | 4 | 3.67 |
| Hippocampus (body) | left | -34 | -18 | -14 | 3.57 |
| Insula | left | -48 | 12 | 6 | 3.57 |
| Superior frontal gyrus | left | -12 | 28 | 58 | 3.33 |
| Middle temporal gyrus | left | -60 | -22 | -6 | 2.77 |
| Middle frontal gyrus | left | -32 | 22 | 44 | 2.75 |

X,Y, and Z coordinates in MNI space, BSR=Bootstrap ratio, *= in the text referred to as ventromedial prefrontal cortex (vmPFC).
